# Omega-3 polyunsaturated fatty acids as adjuvant therapy of colorectal cancer

**DOI:** 10.1007/s10555-018-9744-y

**Published:** 2018-07-03

**Authors:** Milene Volpato, Mark A. Hull

**Affiliations:** Leeds Institute of Biomedical and Clinical Sciences, St James’s University Hospital, University of Leeds, Leeds, LS9 7TF UK

**Keywords:** Cachexia, Chemotherapy, Colorectal cancer, Docosahexaenoic acid, Eicosapentaenoic acid, Omega-3 polyunsaturated fatty acid

## Abstract

The majority of evidence linking anti-colorectal cancer (CRC) activity with omega-3 polyunsaturated fatty acids (O3FAs) has focussed on decreased CRC risk (prevention). More recently, preclinical data and human observational studies have begun to make the case for adjuvant treatment of advanced CRC. Herein, we review latest data regarding the effect of O3FAs on post-diagnosis CRC outcomes, including mechanistic preclinical data, evidence that O3FAs have beneficial effects on efficacy and tolerability of CRC chemotherapy, and human epidemiological data linking dietary O3FA intake with CRC outcomes. We also highlight ongoing randomised controlled trials of O3FAs with CRC endpoints and discuss critical gaps in the evidence base, which include limited understanding of the effects of O3FAs on the tumour microenvironment, the host immune response to CRC, and the intestinal microbiome.

## Omega-3 polyunsaturated fatty acids

Omega-3 polyunsaturated fatty acids (O3FAs) including C20:5ω3 eicosapentaenoic acid (EPA) and C22:6ω3 docosahexaenoic acid (DHA) occur naturally in highest quantities in fish (hence the frequently used term *marine*) [1]. Humans are inefficient at synthesising longer-chain O3FAs from C18:3ω3 α-linolenic acid (LNA) found in vegetable and seed oils. Therefore, the predominant source of EPA and DHA is dietary [2]. O3FAs have well-established anti-inflammatory properties [3] and have found clinical utility for cardiovascular disease prophylaxis and severe hypertriglyceridaemia [4], with emerging evidence that they may be beneficial for treatment of inflammatory bowel diseases [5].

Evidence is also accumulating that O3FAs may have anti-colorectal cancer (CRC) properties. Dietary O3FA intake was originally linked to primary CRC prevention through large epidemiological studies [6]. However, observational human data are now emerging that dietary O3FA status predicts post-diagnosis CRC outcomes and complements a larger body of preclinical evidence that O3FAs may find clinical utility for *treatment* of CRC, as opposed to primary CRC *prevention*. Given the excellent safety and tolerability profile of O3FAs, compared with existing treatment strategies for CRC, aligned with the unmet clinical need for improved adjuvant therapy of CRC, O3FAs have huge potential for use in the advanced post-diagnosis setting. Therefore, this article is restricted to review and interpretation of data supporting treatment of CRC by O3FAs and potential benefit of O3FAs on advanced CRC outcomes.

## Scope of the review

We last reviewed the potential role of O3FAs for prevention and treatment of CRC in 2012 [7]. By then, a large body of preclinical evidence had accumulated to support the case for O3FAs as potential anti-CRC agents, particularly in the prevention setting [7]. A randomised controlled trial in familial adenomatous polyposis patients had demonstrated chemopreventive efficacy of EPA at the early (adenoma) stages of intestinal tumorigenesis [8]. A subsequent review by Komiya et al. in 2013 highlighted the potential of natural compounds such as O3FAs for cancer prevention [9]. Since then, new preclinical and clinical evidence has further strengthened the case for O3FAs as adjuvant therapy for CRC, rather than prevention. This review will focus on data regarding O3FA use for adjuvant CRC therapy in colorectal cancer since our 2012 review, highlighting ongoing studies and gaps to be filled in the evidence base, which may support translation of O3FA therapy into clinical practice. We make a clear distinction between observational data on dietary marine O3FA intake and therapeutic ‘nutraceutical’ supplement use of O3FAs.

## Preclinical data supporting the anti-CRC activity of O3FAs

Multiple mechanisms of actions and molecular targets have been described to explain the anti-inflammatory and anti-cancer activity of O3FAs. These have been reviewed extensively elsewhere [7, 10, 11]. Many of these have been shown to occur in CRC models. They are briefly summarised here before focusing on data that have emerged since 2012 (Table 1).

**Table 1:** Mechanisms shown to contribute to the anti-CRC activity of O3FAs by direct effects on CRC cells

Mechanisms of action	O3FA shown to modulate the pathway	Reports involving CRC models published over the last 5 years
Modulation of cyclooxygenase metabolism	EPA and DHA^a^	[12, 13]
Alteration of lipid raft behaviour	EPA and DHA	
Increase in lipid peroxidation	EPA and DHA	[14]
Induction of pro-apoptotic pathways	EPA and DHA	[15, 16]
Regulation of kinase pathways	EPA and DHA	
G protein-coupled receptor signalling	EPA and DHA	[17]
WNT/ß-catenin pathway modulation	EPA and DHA	
Downregulation of Granzyme B expression	DHA	[18]
Downregulation of P-glycoprotein expression	EPA and DHA	[19]
mTor signalling inhibition	EPA and DHA	[20]

A comprehensive overview of O3FA molecular targets established in preclinical models in a range of cancer types can be found elsewhere [10]

^a^In all cases, EPA and DHA were tested independently

O3FAs can modulate cyclooxygenase (COX) metabolism and reduce production of several prostanoids including prostaglandin (PG) E_2_ in tumours [21, 22], whilst possibly increasing the production of lipid mediators involved in the resolution of inflammation such as lipoxins and resolvins [21, 22], which may have anti-cancer properties [23–25]. Elevated COX-2 expression is found in greater than 90% of CRCs [26–28], associated with high levels of PGE_2_, which drives pro-tumorigenic proliferation, migration, and invasion, but also promotes an immune-suppressive tumour microenvironment beneficial for tumour growth [29, 30].

Proliferation and survival of cancer cells is linked to the activation of signalling pathways from surface molecules, such as cytokine or growth factor receptors [e.g. epidermal growth factor receptor (EGFR)], which transduce signals upon activation *via* protein linked the cytoplasmic membrane and kinase signalling cascades [31]. O3FAs have been shown to incorporate into the plasma membrane of cancer cells, where they alter lipid raft composition and fluidity. This can result in an inhibition of signal transduction, limiting cancer cell survival and promoting apoptosis [32]. O3FAs will also incorporate into non-cancer cell membranes within the tumour microenvironment and potentially alter their phenotype.

O3FAs have also been shown to downregulate other CRC promoting signalling pathways such as the Wnt/ß-catenin pathway [33], the MAPK/ERK pathway [34], and PI3K-PTEN pathway [35, 36].

O3FA accumulation in CRC cells is known to increase lipid peroxidation and cellular oxidative stress [37].

O3FAs can exert anti-CRC activity following their interaction with surface free fatty acid (FFA) G protein-coupled receptors (GPCRs), thereby activating pro-apoptotic signalling [17]. These GPCRs have been shown to be expressed on non-epithelial cells such as adipocytes [38, 39] and macrophages [40], on which activation can alter macrophage polarisation and reduce inflammation that is potentially important for anti-cancer activity of O3FAs.

The respective contribution of these putative diverse mechanisms of action described in *in vitro* and *in vivo* models to potential anti-CRC activity in man is not known and will likely be context-dependent, e.g. tumour type and composition of the microenvironment. Due to the diversity of likely molecular targets of O3FAs, preclinical studies on the potential activity of O3FAs against established CRC, rather than prevention, have focussed on pharmacodynamic endpoints relevant to the hallmarks of cancer such as cell proliferation, apoptosis, and migration.

### O3FAs exert anti-proliferative and pro-apoptotic effects in CRC models

High doses of LNA (over 1 mM) have been shown to reduce cell proliferation, cell adhesion, and the ability of both human (HCT116 and HT29) and mouse (MC38) CRC cell lines to invade matrigel [41]. This study did not indicate the molecular basis of this effect. However, it is unlikely to be COX-2-dependent as mammalian cells are inefficient at converting LNA into EPA or DHA [2]. Similarly, another group investigated the impact of DHA on migration in CRC cell lines and reported that 100 μM DHA could inhibit Granzyme B expression in three human CRC cell lines (HCT116, CSC4, and HT-8), thus reducing their ability to undergo epithelial-mesenchymal transition (EMT) and invade matrigel [18]. The same group has also published similar data in the context of bladder and pancreatic cancer models, suggesting that this mechanism is not tissue-specific [42]. Downregulation of genes related to metastatic behaviour was also highlighted from a list of differentially expressed genes in HT15 CRC xenografts grown in nude mice treated with a DHA-rich diet compared to a control diet (corn oil) for 30 days [43].

In terms of COX-independent activity, both DHA and EPA have been shown to act as ligands for and inhibit cell proliferation *via* GPCRs such as GPR120 [44]. More recently, it has been reported that this interaction leads to the activation of the Hippo signalling pathway in LoVo and HT29 cells *in vitro* [17]. *In vivo*, GPCR activation resulted in a reduction in tumour burden in the azoxymethane (AOM)/dextran-sulphate model in *Balb*/*c* mice fed a 10% fish oil diet [17]. Another study reinforced the link between the anti-cancer effects of O3FAs and oxidative stress, showing that polyunsaturated fatty acids induced apoptosis in human CRC cells (LoVo and RKO cells) *via* the generation of reactive oxygen species and the induction of the caspase cascade [14]. It is notable that this effect was not O3FA-specific as the study reported similar results for both omega-3 (DHA and EPA) and omega-6 (arachidonic acid) FAs at the same concentration of 150 μM *in vitro* [14]. The same authors published a parallel study using the same models showing that the effect of polyunsaturated fatty acids on cell proliferation and lipid mediators [12]. The data highlight the context-dependent manner of the mechanisms of action of EPA and DHA as both reduced cell proliferation in each cell line, but 150 μM DHA induced an increase in PGE_2_ and lipoxin A4 (LXA4) in levels in LoVo cells, but not in RKO cells, with the opposite result obtained when treating cells with 150 μM EPA [12].

The presence of cancer stem cells within a tumour mass has been linked to resistance to radiotherapy and chemotherapy. More than one research group has investigated whether O3FAs could exert their anti-cancer activity cancer stem-like cells within the tumour mass. De Carlo et al. (2013) used COLO320 DM cells, which grow as a mixed population of CD133^−^ cells and CD133^+^ cancer stem-like cells, as an *in vitro* model of a mixed tumour cell population. They established that doses of EPA, which are comparable to that achieved in human plasma, reduced proliferation of “standard” cancer cells (CD133^−^) but not CD133^+^ stem cell-like cancer cells. However, in the presence of EPA, there was a change in the ratio of “standard” to stem cell-like markers with a reduction in CD133 expression level and an increase in epithelial marker expression such as MUC2 [45]. Another study suggested that O3FAs could affect both CD133^+^ and CD133^−^ cells: EPA and DHA were shown to reduce cell proliferation in SW620 monolayer and 3D cultures that display a stem cell-like phenotype [46]. Using the LS174T cell line, which is considered a model of CRC initiating cells with stem-like properties, Sam et al. (2016) showed that both EPA and DHA (at concentrations between 50 and 150 μM) reduced LS174T cell growth in a time- and dose-dependent manner. The authors proposed that O3FAs decreased survivin expression and induced caspase-3 activation to promote cell death [16].

Although data continue to accumulate supporting the hypothesis that O3FA are good candidate compounds for the treatment of CRC, in the era of personalised cancer therapy, there remains a lack of studies investigating predictive markers of response to O3FA in CRCs in a translational setting. One study reported that EPA exposure decreased C-C motif chemokine ligand 2 (CCL2) production and expression of its receptor C-C chemokine receptor 2 (CCR2) expression in human HCA-7 and mouse MC38 CRC cells in a dose-dependent matter *in vitro* [13]. These results were confirmed *in vivo* using a MC38 xenograft model and were then translated into the clinical setting with demonstration that changes in plasma CCL2 levels in EPA-treated CRC liver metastasis patients [47] were associated with a specific tumour gene expression profile and may predict patient CRC outcomes [13].

### Novel formulations to improve O3FA efficacy

O3FA are commercially available in various formulations: as the FFA, conjugated to ethyl esters (EE), as a triglyceride (TG), or as phospholipids. Only a limited number of studies have investigated novel formulations to improve the anti-cancer effect of O3FAs.

In 2013, Morin et al. reported the impact of O3FA conjugation as the mono-glyceride as opposed to FFA, EE, or TG on the O3FA incorporation and anti-cancer activity. They showed that monoglyceride-conjugated O3FAs were more efficiently incorporated in HCT116 cells than as the FFA form, but also displayed greater anti-proliferative activity in this model, potentially *via* lipoxygenase and CYP450 metabolism [48]. Docosapentaenoic acid (DPA) was especially promising in this form and was also shown to inhibit HCT116 tumour growth *in vivo* [48]. The same group went on to establish the anti-inflammatory properties of these agents in non-cancer disease models such as cystic fibrosis [49]. More recently, they have demonstrated that DHA-monoglyceride could be used to potentiate carboplatin anti-cancer activity both *in vitro* and *in vivo* in A549 and H1299 lung cancer models [50]. It remains to be seen whether these results can be translated to the CRC setting, in which patients more likely receive oxaliplatin chemotherapy.

More recently, encapsulation of LNA or DHA in liposomes with the anti-oxidant polyphenol resveratrol was shown to increase incorporation of the O3FA in HT29 cells [51]. Serini and colleagues also suggested that this liposome formulation lead to an increased conversion of LNA into EPA/DHA [51]. They also reported increased cell growth inhibition in HT29 and HCT116 cells treated with O3FA-loaded liposomes compared to the FFA equivalent, associated with a reduction in proliferation rate, but no increase in apoptosis induction [51]. This formulation requires validation in *in vivo* models to ascertain its bioavailability and efficacy.

### Combination O3FA treatment with chemotherapies and other nutraceuticals

Cancer therapies are rarely administered as single agents. A combination of agents often allows dose reduction of one or more agents in order to mitigate the risk of cumulative side effects. In this context, O3FAs represent strong potential for adjuvant therapy given their low toxicity profile, allowing the use of other agents at more effective doses. A previous study demonstrated that EPA and DHA can modulate cholesterol synthesis and as a result downregulate the expression of the efflux pump, P glycoprotein, in a doxorubicin-resistant variant of HT29 cells. Gelsomino et al. suggested this reduction in drug efflux pump expression could be significant when using O3FAs in combination with standard chemotherapies limited by such detoxifying mechanisms [19].

- Improved response to standard-of-care chemotherapy

Multiple studies have analysed whether O3FAs could improve response to chemotherapeutic agents routinely used in the treatment of CRC. Vasudevan et al. demonstrated a synergistic anti-cancer effect between EPA and a regimen of 5-fluorouracil (5-FU) and oxaliplatin *in vitro* and *in vivo* against HT29 and HCT116 CRC models [52]. Several other *in vivo* studies have demonstrated that O3FAs can both potentiate 5-FU anti-cancer activity (reduction of tumour burden, increased apoptosis, and DNA damage) and also reduce 5-FU-related toxicity [53–55]. In 2017, a study showed that 5-FU and irinotecan treatment can lead to impaired lipid storage in rat models, resulting in loss of O3FA in tissues. The authors hypothesised that combining O3FA with standard chemotherapy regimens could help restore lipid stocks, thus potentially limiting 5-FU-associated side effects [56].

Pichard and colleagues have focussed on the potential combination of O3FAs with standard CRC treatment modalities for over a decade. They have shown that O3FAs have the potential to radio-sensitise cancer cells, demonstrating that radio-resistant HT29 cells became responsive to radiation when treated with O3FA, and DHA in particular [57]. An additive cytotoxic effect was also observed in radio-resistant LS174T (stem cell-like) CRC cells. The proposed mechanism for this enhanced response was an increase in lipid peroxidation products within the cells [57]. The same group has also investigated the impact of O3FAs on the anti-CRC activity of 5FU, oxaliplatin, and irinotecan on HT29 and LS174T cells. They reported an increase in apoptosis when combining these agents with a fish oil emulsion containing 20.6 g/L of EPA and 19 g/L of DHA [15].

In the preclinical study of EPA activity on CD133^+^ COLO320DM cells [45], low-dose EPA (25 μM) exposure not only sensitised CD133^−^ COLO 320 DM cells to both 5-FU and oxaliplatin but also increased the sensitivity of the CD133^+^ stem-like cell population to 5-FU [45]. Likewise, an increased sensitivity to 5-FU and mitomycin C was observed in SW620 human CRC cells when combined with low-dose O3FA [46].

Aside from standard chemotherapies, DHA has been shown to enhance TRAIL-induced apoptosis in SW620 CRC cells but not normal colorectal NCM460 epithelial cells, suggesting that a combination of O3FAs with an agent targeting this apoptosis pathway should be investigated as a novel anti-cancer therapy [58].

- Combination of O3FAs with other nutraceuticals

Combinations with other naturally occurring compounds such as curcumin, which have been previously considered for cancer prevention strategies, are now being tested for therapeutic interventions in the advanced cancer setting. A novel potential mechanism of action was highlighted in an *in vitro* study, which showed that a combination of EPA with two natural phytochemicals, grape seed extract and epigallocatechin-3-gallate (found in green tea), could inhibit mTOR as effectively as rapamycin in SW480 and HCT116 cells [20]. Kim et al. used the AOM model to induce colonic carcinogenesis and establish the impact of curcumin and O3FA on cancer stem cell survival. They showed that the combination of curcumin and O3FA resulted in an increase in apoptosis, as well as a reduction in nuclear ß-catenin in Lgr5^+^ colonic stem cells, within aberrant crypts [59]. Another study combined DHA with butyrate, a short-chain fatty acid used by colonocytes for energy production with known histone deacetylase inhibitory properties, which enhanced apoptosis compared with butyrate alone through increased downregulation of promoter methylation of several pro-apoptotic genes such as Tnfrsf25 [60].

## Clinical data on the anti-CRC activity of O3FAs

Despite an increasing body of evidence from preclinical studies that O3FAs may have anti-CRC activity in the post-CRC diagnosis setting, human intervention studies of the effect of O3FA supplementation on CRC outcomes (primary CRC diagnosis, recurrence, and CRC-related mortality) have been limited, to date. The majority of clinical data linking post-diagnosis CRC outcomes with O3FA intake continues to be observational.

### Observational data

A study of the relationship between dietary marine O3FA (EPA, DHA, and DPA) intake and post-CRC diagnosis outcomes using the well-established Nurse’s Health Study and Health Professionals’ Follow-up Study cohorts demonstrated that those individuals with highest O3FA intake (measured by food frequency questionnaire) had reduced risk of CRC mortality after primary CRC diagnosis [61]. Moreover, those individuals who increased marine O3FA intake after CRC diagnosis had decreased risk of CRC mortality (HR 0.30, 95% CI 0.14–0.64) [61]. The same group has also described that high marine O3FA intake is associated with lower risk of primary CRC that is proximal rather than distal [62], demonstrates high microsatellite instability (MSI-H) rather than being microsatellite stable (MSS) [63], and with a high FOXP3-positive (regulatory) T cell infiltrate [64]. These data suggest that O3FAs may act preferentially on MSI-H CRCs by promoting host anti-tumour immuno-surveillance mechanisms. Similar biomarker stratification should be applied to future clinical studies of the effects of O3FAs on CRC recurrence in order to determine whether O3FAs act preferentially on specific CRC subtypes.

More recently, the relationship between marine O3FA intake and survival in the CALGB 89803 randomised trial of adjuvant chemotherapy for completely resected stage III CRC (*n* = 1264) has been investigated retrospectively [65]. Patients in the highest quartile of O3FA dietary intake had increased disease-free survival (DFS) compared with the lowest quartile (HR 0.72 [0.54–0.97]). This relationship appeared to be stronger for individuals with high CRC COX-2 expression [65].

Epidemiological evidence that O3FA supplementation, rather than dietary O3FA intake and/or tissue O3FA status, is associated with improved CRC outcomes remains scanty. The VITAL cohort study collected data on O3FA supplement use, as well as dietary O3FA intake, unlike the vast majority of epidemiological studies investigating the link between O3FA intake and CRC risk, which have not attempted to quantify fish oil or purified O3FA supplement use [6]. It demonstrated that fish oil supplement users (≥ 4 days per week for ≥ 3 years) had 49% decreased CRC risk compared with non-users [66].

### Intervention trials

The only randomised trial of purified O3FA treatment in patients with metastatic CRC that has been reported is the EMT study [47]. The EMT study was a phase II double-blind, randomised, placebo-controlled trial of EPA, in the FFA form, 2 g daily before surgery in patients (*n* = 88) undergoing liver resection of CRC liver metastases. Although the primary endpoint was the tumour Ki67 proliferation index, overall survival (OS) and DFS were specified exploratory endpoints. In the first 18 months after CRCLM resection, EPA-treated individuals obtained OS and DFS benefit compared with placebo (HR for OS 0.40 [0.16–1.0], *P* = 0.05) [47]. This preliminary observation from a “window” trial of limited O3FA use prior to metastasis surgery has led to an ongoing phase III randomised trial of EPA (4 g daily in the EE form) in patients undergoing liver resection surgery for CRC liver metastasis (the EMT2 trial), in which subjects are randomised to EPA or placebo at least 2 weeks before surgery and continue medication long-term, with progression-free survival (PFS) as the primary endpoint and OS as the key secondary endpoint (ClinicalTrials.gov; NCT03428477).

There have been 11 studies of the perioperative use of O3FA-containing enteral and parenteral nutrition supplements in CRC patients (overall *n* = 694), which are the subject of a recent meta-analysis [67]. Overall, O3FA-containing perioperative nutrition was associated with reduced post-operative complications, lower pro-inflammatory cytokine levels, and reduced hospital stay [67]. In the largest of the randomised trials, Sorensen and colleagues have reported that an O3FA-containing (EPA 2 g and DHA 1 g per day) oral nutritional supplement given 7 days before and 7 days after elective CRC surgery had no effect on infectious or non-infectious post-operative complications [68]. Long-term CRC outcomes were not reported in this study [68]. There has been no study of a purified O3FA formulation in the setting of primary CRC surgery. However, the EMT study confirmed that EPA treatment is safe in the context of CRC liver resection surgery with no excess of bleeding despite the modest anti-platelet activity of O3FAs [47].

Two randomised trials of O3FA supplementation are underway that have secondary CRC endpoints. The ASCEND trial (NCT00135226) is a 2 × 2 factorial study of long-term (median 7.5 years) O3FAs (840 mg EPA/DHA EE daily) and aspirin (100 mg daily) treatment for prevention of cardiovascular and cerebrovascular events in patients with diabetes (*n* = 15,480). Cancer outcomes are a secondary endpoint, with the ability to continue with post-trial follow-up. VITAL (NCT01169259) is a 2 × 2 factorial study of the same dose and formulation of O3FAs (also 840 mg EPA/DHA EE) and vitamin D3 (2000 IU daily) in 25,871 participants, in which CRC is a specified secondary outcome [69].

### CRC cachexia

Despite the fact that several systematic reviews have failed to demonstrate a beneficial effect of O3FAs on cancer cachexia, there is still interest in potential benefit of EPA on cachexia related to advanced CRC given the known anti-inflammatory properties of EPA [70] and the current relatively weak evidence base for anti-cachexic activity reliant on small, heterogeneous studies [71, 72]. The EMT2 trial will provide important data on the effect of EPA on OS after CRC progression following CRC liver metastasis surgery. Assessment of paravertebral sarcopenia by routine CT imaging during intervention with EPA or placebo is a planned exploratory analysis (ClinicalTrials.gov; NCT03428477).

### O3FA and CRC chemotherapy

The effect of O3FA supplementation on either efficacy or tolerability of traditional chemotherapy for CRC has not been subjected to definitive clinical evaluation despite promising preclinical data on combination therapy reviewed above and encouraging preliminary clinical data. Building on small studies of the effect of fish oil supplementation (EPA 360 mg and DHA 240 mg per day) for 9 weeks on laboratory nutritional parameters in patients with stage II–IV CRC undergoing chemotherapy [73, 74], this group retrospectively analysed CRC outcomes and administration of chemotherapy (a variable combination of capecitabine, oxaliplatin, 5-fluorouracil, and leucovorin) in 30 individuals randomised to fish oil or no supplementation [75]. There was longer progression-free survival in those randomised to fish oil, but no difference in number of chemotherapy cycles administered, number of days of chemotherapy, or delays/interruptions in chemotherapy cycles [75]. These preliminary data should prompt detailed randomised trial evaluation of the effect of higher-dose O3FAs on tolerability of standard chemotherapy for CRC, as well as on longer-term CRC outcomes in the context of adjuvant chemotherapy.

Voest and colleagues first reported that platinum-based chemotherapeutics induced COX-1-dependent production of so-called platinum-induced fatty acids (PIFAs), including the O3FA hexadecatetraenoic acid (C16:4ω3), which induce resistance to chemotherapy in mice, in 2011 [76]. However, although PIFAs are detectable in humans and complex fish oil supplements contain PIFAs [77], the relevance of PIFAs to CRC chemotherapy in man and their impact related to individual O3FA status (dietary and/or tissue levels) or adjuvant use of purified O3FA formulations remains unclear. The Dutch group has recently published a phase I trial of the use of the COX-1 inhibitor indomethacin with cisplatin/oxaliplatin reporting that indomethacin use was associated with reduced levels of one PIFA (12*S*-HHT), but not the other (C16:4ω3), in patients with CRC or oesophageal cancer [78].

Preclinical data also suggest that O3FAs inhibit EGF receptor family signalling and could augment activity of anti-EGF receptor agents [32, 79]. Dietary O3FA intake and/or tissue O3FA levels should be analysed retrospectively in previous trials of cetuximab and panitumumab for wild-type RAS metastatic CRC in order to make the case for definitive clinical evaluation of O3FA in that therapeutic context.

The intriguing observation that dietary O3FA intake is associated with reduced CRC risk for those tumours with MSI-H features and a high regulatory T cell population [63, 64] suggests that O3FAs may have anti-CRC activity by promoting the host anti-tumour immune response. The hypothesis that O3FAs may augment the therapeutic response to immune checkpoint inhibitor therapy either in MSI-H or MSS CRCs warrants testing.

### O3FA and the intestinal microbiota

There is currently much interest in the effects of O3FA supplementation on the human intestinal microbiota. This is likely to be a relevant potential mechanism for reduction in CRC risk in a primary prevention setting, but may also be relevant to the possible use of O3FA as adjuvant treatment of CRC, given the recognition that the intestinal microbiota may modulate response to cancer therapy and modify toxicity [80].

A series of rodent experiments have delineated that dietary O3FA supplementation alters the intestinal microbiota in favour of “beneficial” genera such as *Bifidobacterium* and *Lactobacillus* [81, 82]. A recent randomised crossover trial of 4 g mixed O3FAs per day in healthy middle-aged volunteers did not report any change in overall bacterial diversity but did show increased abundance of several short-chain fatty acid-producing genera, including *Bifidobacterium*, during O3FA supplementation [83]. An observational study of middle-aged to elderly women has reported that serum O3FA levels correlate with intestinal microbiome diversity and abundance of specific bacteria, strongest for the butyrate-producing *Lachnospiraceae* family [84]. Changes in the intestinal microbiome should be investigated in subsequent studies of the effects of O3FAs on chemotherapy and advanced CRC outcomes.

A recent double-blind, randomised, placebo-controlled trial investigated the effect of combination treatment with O3FA (EPA and DHA each 700 mg daily) and a probiotic supplement on tolerability of capecitabine/oxaliplatin chemotherapy and inflammatory markers [85]. Supplementation with combined probiotic and O3FA preparations was associated with improved overall quality of life and reduced chemotherapy-induced symptoms including diarrhoea and fatigue [85]. Mechanistic studies on the intestinal microbiome or tissue O3FA incorporation were not reported in this study.

## Summary

**Fig. 1:**
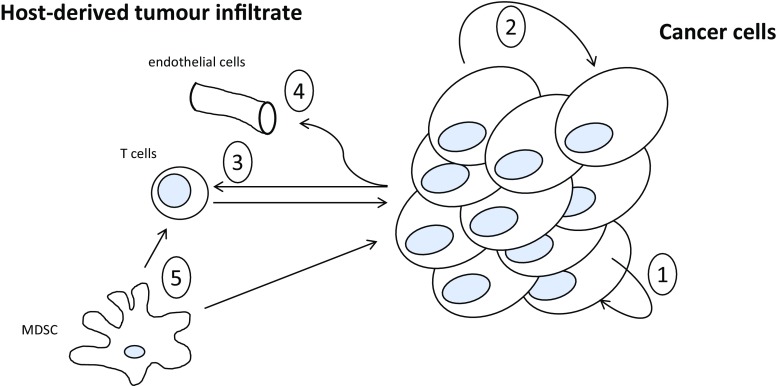
Potential effects of O3FAs on the crosstalk between cancer cells and the host-derived cell infiltrate in the tumour microenvironment. O3FAs are believed to exert their anti-cancer activity through multiple mechanisms including direct effects on cancer cells, but also inhibition of paracrine signalling between cancer cells themselves or neighbouring stromal cells (including the host innate and acquired immune cell infiltrate, endothelial cells, and fibroblasts). In addition, direct effects on the stromal cell infiltrate may alter pro- or anti-tumorigenic activity of these cells. An example is provided by inhibition of COX-dependent PGE_2_ production, which occurs in CRC cells themselves, thereby reducing autocrine (1) and paracrine (2) cell proliferation signalling in malignant epithelial cells, but could also abrogate the immuno-suppressive activity of PGE_2_ on host immune surveillance (3) and impair angiogenesis (4). Moreover, O3FAs may inhibit PGE_2_ production directly by tumour stromal cells, including myeloid-derived suppressor cells (MDSCs) (5)

Over the past 5 years, the body of laboratory and preclinical evidence supporting a role for O3FAs for treatment of CRC has continued to grow. However, translation into the clinic *via* randomised clinical evaluation has not occurred to date. There remain several gaps in the evidence base for adjuvant O3FA therapy of CRC that are hampering translation (Table 2). For example, there are no comprehensive comparative analyses of the effect of EPA alone *versus* DHA alone *versus* an O3FA mix against CRC (Table 2), despite their known differential modes of action *in vitro* [10]. Moreover, the majority of preclinical data focus on the direct effect of O3FA on cancer cells when O3FAs have strong anti-inflammatory properties, which may also alter the tumour microenvironment (Table 2). For example, O3FAs can reduce PGE_2_ production by cancer cells (Fig. 1), thereby inhibiting cancer cell proliferation, but reduced PGE_2_ in the tumour milieu will also abrogate PGE_2_-dependent immuno-suppression [86] and impair angiogenesis [87]. In addition, O3FAs act on tumour stromal cells, such as myeloid-derived suppressor cell (MDSCs) directly, reducing stromal cell PGE_2_ production [88], thus abrogating further the pro-proliferative and immuno-suppressive activity of intra-tumoral PGE_2_ (Fig. 1).

**Table 2:** Some proposed future directions for research into O3FAs as adjuvant CRC therapy

Future research directions driven by critical gaps in knowledge	
Defining the contribution of the effects of O3FAs on tumour immunology as opposed to direct effects on CRC cells Understanding the mechanisms of action of EPA compared with DHA and whether combination O3FA therapy provides additional benefit over single O3FA use Establishing predictive CRC biomarker(s) of O3FA response based on better understanding of mechanism of action, e.g. tumour COX expression, GPCR expression, and O3FA levels Better understanding of the effect of O3FAs on efficacy and toxicity of standard chemotherapy, as well as new biologic therapies, including intestinal microbiome research Adequately powered study of purified O3FA formulations on survival and cachexia biomarkers in advanced CRC

Ongoing randomised trials, including the EMT2 trial, may bolster the case for O3FA use in the advanced CRC setting and should provide much needed mechanistic clinical data that support laboratory findings, including intestinal microbiome analysis, as well as tumour and blood immuno-phenotyping (Table 2). We predict that there will be particular focus on the effects of O3FAs on tumour immunology (as opposed to direct effects on CRC cells themselves) and also whether O3FAs augment activity (and decrease toxicity), of both traditional and newer chemotherapies for CRC. Increased emphasis on molecular subtypes of CRC will lead to stratified analysis of CRC outcomes related to consensus molecular subtypes [89]. Given the large number of potential mechanisms of the anti-CRC activity of O3FAs (including potential anti-cachexia properties) that have been described, a more pragmatic approach to clinical evaluation of O3FA therapy, with “bolt-on” mechanistic studies, is required in order to translate preclinical findings into therapeutic use of O3FAs in the clinic (Table 2).
